# Agricultural Selection of Wheat Has Been Shaped by Plant-Microbe Interactions

**DOI:** 10.3389/fmicb.2020.00132

**Published:** 2020-02-06

**Authors:** Andrzej Tkacz, Francesco Pini, Thomas R. Turner, Eloïne Bestion, James Simmonds, Phil Howell, Andy Greenland, Jitender Cheema, David M. Emms, Cristobal Uauy, Philip S. Poole

**Affiliations:** ^1^Department of Plant Sciences, University of Oxford, Oxford, United Kingdom; ^2^John Innes Centre, Norwich Research Park, Norwich, United Kingdom; ^3^National Institute of Agricultural Botany, Cambridge, United Kingdom

**Keywords:** rhizosphere, microbiota, wheat, polyploidy, crop domestication, Triticaeae

## Abstract

The influence of wheat (modern wheat, both bread and pasta, their wild ancestors and synthetic hybrids) on the microbiota of their roots and surrounding soil is characterized. We isolated lines of bread wheat by hybridizing diploid (*Aegilops tauschii*) with tetraploid *Triticum durum* and crossed it with a modern cultivar of *Triticum aestivum*. The newly created, synthetic hybrid wheat, which recapitulate the breeding history of wheat through artificial selection, is found to support a microbiome enriched in beneficial Glomeromycetes fungi, but also in, potentially detrimental, Nematoda. We hypothesize that during wheat domestication this plant-microbe interaction diminished, suggesting an evolutionary tradeoff; sacrificing advantageous nutrient acquisition through fungal interactions to minimize interaction with pathogenic fungi. Increased plant selection for Glomeromycetes and Nematoda is correlated with the D genome derived from *A. tauschii*. Despite differences in their soil microbiota communities, overall wheat plants consistently show a low ratio of eukaryotes to prokaryotes. We propose that this is a mechanism for protection against soil-borne fungal disease and appears to be deeply rooted in the wheat genome. We suggest that the influence of plants on the composition of their associated microbiota is an integral factor, hitherto overlooked, but intrinsic to selection during wheat domestication.

## Introduction

**FIGURE 1:**
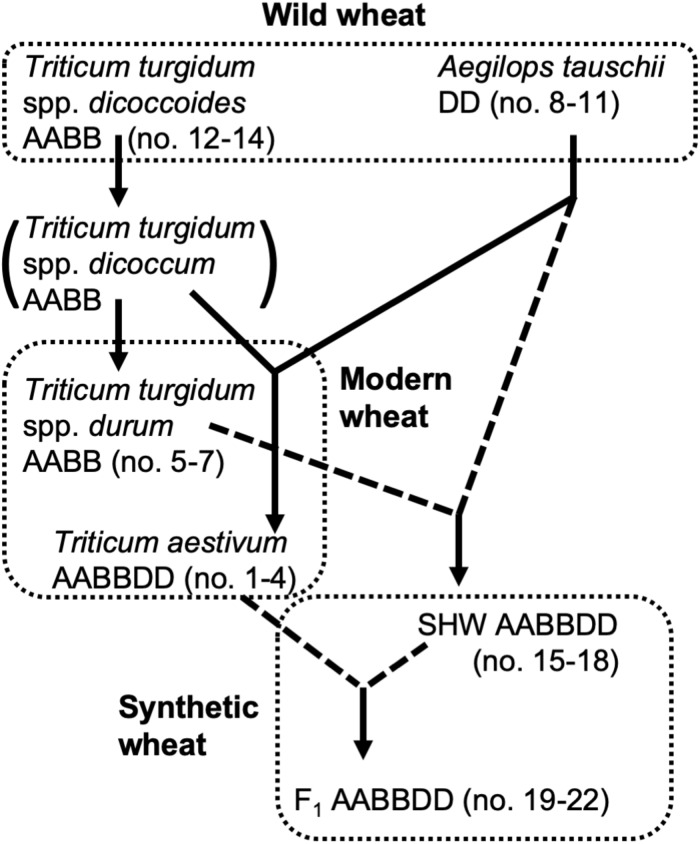
Genetic relationship of wheat species used in this study. Dotted lines indicate hybridization and crossing performed for this study.

Selection of domesticated wheat varieties for improved yield has reduced their genetic diversity (Doebley et al., 2006; Purugganan and Fuller, 2009) which may be a factor which will hinder their future sustainability when faced with (re-) emerging pathogens and climate change. Increasing genetic diversity of wheat, facilitated by advanced genomics technologies (Bevan and Uauy, 2013), is therefore seen as key to sustaining world food supplies (Jonas and de Koning, 2013). Wild tetraploid wheat, *Triticum turgidum* spp. *nicocodines* originated approximately 400,000 years ago from the polyploidization of two closely related diploid species (Dvorak et al., 2006). Around 7,000–9,500 years ago, hexaploid wheat *Triticum aestivum*, one of the most important crops cultivated today, emerged as a result of the hybridization of the diploid *Aegilops tauschii* with a wild tetraploid grass *T. turgidum* (spp. *dicoccum*) (Dubcovsky and Dvorak, 2007) (summarized in Figure 1).

In addition to yield-based selection, primitive agriculture was also strongly affected by invasive plants, animal, fungal and bacterial pests, climate stress and, although perhaps not previously apparent, on the plants’ ability to control its soil and root microbiota. Effects of crop domestication on plant microbiotas have been recently proposed, suggesting that modern cultivars may have lost some genetic features required for recruitment and nurturing specific microorganisms (Bulgarelli et al., 2013; Perez-Jaramillo et al., 2015, 2018). Domestication of maize (*Zea* spp.) has been shown to influence bacterial communities of the rhizosphere and root endosphere (Johnston-Monje and Raizada, 2011; Bouffaud et al., 2012). To understand how the genomic composition of wheat influences the recruitment of microorganisms associated with roots, the microbiota structure of four different wheat species, differing both in genomic content and in domestication and selection history have been analyzed (Figure 1). These include numerous lines of each of the following; the diploid wild *A. tauschii* (DD genome), tetraploid progenitor species *T. turgidum* spp. *dicoccoides* (AABB), domesticated pasta wheat *T. turgidum* spp. *durum* (AABB), and the hexaploid bread wheat *T. aestivum* (AABBDD) (Table 1). Furthermore, by developing synthetic hexaploid wheat (SHW) lines (AABBDD) which recreate the hybridization event between *A. tauschii* and *T. durum*, we recapitulated, as far as is possible, the evolution of modern bread wheat (Figure 1). Understandably, the lines used for hybridization have been under natural and also human selection and hence are different from the wild plants from 7,000 to 9,500 years ago. In order to further investigate interactions between the wheat genome and soil microbiota, a set of F_1_ hybrids between synthetic hexaploid wheat (SHW) and *T. aestivum* cv. Paragon was bred (Table 1). These F_1_ plants are hybrids in which one intact set of chromosomes (ABD) comes from the modern elite cultivar Paragon (domesticated and later artificially selected through agricultural usage), while the other set of chromosomes comes from a newly created SHW line (Figure 1 and Table 1).

**TABLE 1:** Wheat species and analysis performed.

	Plant	Line	History	Genome	D-genome	No. of biol. reps.	Rhizosphere	Root

Modern wheat								
**Bread wheat**								
1	*T. aestivum*	Paragon	elite	AABBDD	artificially selected D	9	P/E/F/O	P
2	*T. aestivum*	Rialto	elite	AABBDD	artificially selected D	9	P/E/F/O	P
3	*T. aestivum*	Avalon	elite	AABBDD	artificially selected D	10	E/F/O	
4	*T. aestivum*	Watkins-1190032	landrace	AABBDD	artificially selected D	9	E/F/O	
**Pasta wheat**								
5	*T. durum*	Biensur	domesticated	AABB	no D	8	P/E/F/O	P
6	*T. durum*	Hoh-501	domesticated	AABB	no D	8	P/E/F/O	P
7	*T. durum*	Kronos	domesticated	AABB	no D	9	E/F/O	

**Wild wheat**								
**Goat grass**								
8	*A. tauschii*	Ent 392	wild	DD	wild D	8	P/E/F/O	P
9	*A. tauschii*	Ent 336	wild	DD	wild D	10	P/E/F/O	P
10	*A. tauschii*	Ent 088	wild	DD	wild D	7	E/F/O	
11	*A. tauschii*	WX895	wild	DD	wild D	8	E/F/O	
**Emmer wheat**								
12	*T. dicoccoides*	TTD140	wild	AABB	no D	6	P/E/F/O	P
13	*T. dicoccoides*	DIC70	wild	AABB	no D	10	P/E/F/O	P
14	*T. dicoccoides*	DIC63	wild	AABB	no D	8	E/F/O	
**Synthetic wheat**								
15	SHW	NIAB SHW-023	Hoh-501, WX895	AABBDD	wild D	10	E/F/O	
16	SHW	NIAB SHW-041	Biensur, Ent392	AABBDD	wild D	10	P/E/F/O	P
17	SHW	NIAB SHW-054	Hoh-501, Ent336	AABBDD	wild D	10	P/E/F/O	P
18	SHW	NIAB SHW-055	Hoh-501, Ent088	AABBDD	wild D	10	E/F/O	
19	F1	F1 line 1	Paragon × SHW-023	AABBDD	wild D/artificially selected D	7	E/F/O	
20	F1	F1 line2	Paragon × SHW-041	AABBDD	wild D/artificially selected D	6	E/F/O	
21	F1	F1 line3	Paragon × SHW-054	AABBDD	wild D/artificially selected D	7	E/F/O	
22	F1	F1 line4	Paragon × SHW-055	AABBDD	wild D/artificially selected D	10	E/F/O	
	unplanted	bulk soil control				8	P/E/F/O	P

*The two last columns list the experiments performed for each wheat line, P, prokaryotes; E, eukaryotes; F, fungi; and O, oomycetes. Footnote about qPCR Rhizosphere P/E taken from Table as a column which was the same in all.*

Previously, we have shown that plants are able to modify their rhizosphere, even down to the ratio of eukaryotes to prokaryotes they contain. *Pisum sativum* (pea) and *Avena strigosa* (oat) significantly increase the relative abundance of fungi and other microbial eukaryotes relative to bulk soil, to approx. 21%, while *T. aestivum* cv. Paragon rhizosphere soil has a low ratio of eukaryotes to prokaryotes, similar to that found in bulk soil (Turner et al., 2013). It is unclear why *T. aestivum* differs from these two other plant species, although potential explanations include factors such as root architecture and pattern of root exudation. These factors are determined, at least in part, by the plant genomic content which has been influenced by plant breeding, artificial selection history and ploidy.

The goal of this study was to test whether genetic-based breeding of crops influences the microbial community and whether the microbiota is a hitherto hidden factor in crop domestication and artificial selection during both early agriculture and modern breeding selection. We conclude that wheat species, despite sharing a vast core microbiota, do select for specific prokaryotic and eukaryotic taxa, however, none of the wheat lines analyzed significantly modify the ratio between eukaryotes and prokaryotes in their microbiota. We unravel the significance of the D genome derived from wild *A. tauschii* in supporting Glomeromycetes fungi, many of them being able to provide P to the plants, and, potentially pathogenic, Nematoda species. We hypothesize that minimizing this plant-fungal interaction proved a desirable property during domestication as agricultural practices were able to provide plants with external P, but resistance to nematodes was a desirable trait which was selected for.

## Materials and Methods

### Experimental Design

The soil was collected from a naturally grassed and unfertilized part of John Innes Centre’s Church Farm, Bawburgh, Norfolk, United Kingdom (52°37′39.35″N, 1°10′43″E). Covering vegetation was stripped off and soil collected from a depth of 10–30 cm. Soil was air-dried and sieved to remove stones and roots. Chemical analyzes were performed by Macaulay Soils (James Hutton Institute, Aberdeen, United Kingdom) and have been reported previously (Tkacz et al., 2015). Germplasm used is shown in Table 1 and includes four lines of *T. aestivum* (no. 1–4), three lines of *T. turgidum* spp. *durum* (no. 5–7), four lines of *A. tauschii* (no. 8–11), and three lines of *T. turgidum* spp. *dicoccoides* (no. 12–14). Four SHW lines (no. 15–18) were generated from each of the *A. tauschii* lines (no. 8–11) with one of two different *T. durum* lines (no. 5 or 6). Finally, each of the SHW lines (no. 15–18) was crossed with elite *T. aestivum* cv. Paragon (no. 1) to produce four F_1_ lines (no. 19–22) (Table 1 and Figure 1). Seeds were washed but not surface-sterilized (as many of *A. tauschii* seeds could not tolerate either bleach or ethanol treatment) and were germinated on MS agar for 2 days at room temperature in the dark. Only plants from clean MS plates (without any bacterial or fungal contamination) were used as a seedling source. Seedlings were planted in 200 ml pots filled with soil and covered with perlite (to prevent algae growth), before being grown for 6 weeks in a glasshouse. Plants were watered using a pipe going down through the perlite layer. A single plant per pot was grown and its root and the rhizosphere used as a biological replicate for a given species line (*n* = 6–10 replicates, depending on the line – Table 1). All plants were harvested at the heading/flowering stage (Zadok growth stage 59) within a single week. As a control (referred to in text and figures as bulk soil), plant-free pots were established and given the same treatment as pots with plants. In this study, for practical reasons, a single soil was used, however we have already established in the previous studies that our Bawburgh soil contains a typical soil microbiota community (Tkacz et al., 2015, 2018), and recently have shown that many plant species, including wheat, behave in a very similar way regarding microbiota structuring in different soils [i.e., Bawburgh soil (used in this work) and soil from Wytham, Oxford, United Kingdom] (Tkacz et al., 2020). In addition, the work of Bulgarelli et al. (2012) shows that Arabidopsis selects a similar root microbiota whether the soils are sampled in Germany or United States. However, in soils of low or high pH and/or contaminated with heavy metals, plants can have a strong influence on microbiota (Griffiths et al., 2011; Zadel et al., 2019).

### DNA Extraction, PCR, and qPCR Conditions

Plant harvest and DNA isolation were performed as previously described (Turner et al., 2013). Briefly, plants were harvested, and loosely attached soil was discarded by shaking. Stems were removed, and rhizosphere soil was washed off roots by vortexing them in a 50 ml tube with distilled water. Roots were removed, and soil was collected by centrifugation (rhizosphere sample). Collected roots were washed again until visually clean from any soil debris and then crushed using a pestle in a mortar filled with liquid nitrogen and silica sand (root sample). DNA from each rhizosphere (300 mg) and root sample (300 mg) was extracted according to the specification of ZYMO research DNA isolation kit D6001 (Irvine, CA, United States), quantified with the Qubit 2.0 (Invitrogen, Carlsbad, CA, United States) and standardized to the concentration of 5 ng/μl. Each sample (199 samples in total) was PCR-amplified with four sets of primers: prokaryotic primers targeting V4 region of 16S rRNA gene; 515F 5′-GTGCCAGCMGCCGCGGTAA-3′ and 806R 5′-ATTAGAWACCCBDGTAGTCC-3′ (Caporaso et al., 2012) amplifying both bacterial and archaeal DNA; eukaryotic primers targeting a fragment of 18S; F1427 5′-TCTG TGATGCCCTTAGATGTTCTGGG-3′ and R1616 5′-GCGGT GTGTACAAAGGGCAGGG-3′ (van Hannen et al., 1998), able to target a broad range of microbial eukaryotes, such as algae, diatoms, animals, plants, protists, and fungi. There are at least 439 primer pairs designed for fungal and/or eukaryotic microorganisms’ detection (Banos et al., 2018). Hence, we decided to use the eukaryotic 18S rRNA primers which we have previously successfully used to amplify a broad range of soil eukaryotic microorganisms and based on the quantitative spike-in approach we have established that these primers allow for an order of magnitude higher detection of fungal presence in soil samples than a standard ITS primer set (Tkacz et al., 2018), which we consider to be an important feature for the quantitative PCR (qPCR) approach undertaken in this study (explained later in the “Materials and Methods” section). For fungi specific detection we used primers targeting the intergenic region between 18S and 5.8S rRNA genes; ITS1F 5′-CTTGGTCATTTAGAGGAAGTAA-3′ and ITS2 5′- GCTGCGTTCTTCATCGATGC-3′ (Cardinale et al., 2004) (here called fungal ITS1) normally limited to amplification from Ascomycota and Basidiomycota fungal species (Buee et al., 2009) and for oomycetes detection we used primers targeting the intergenic region between 18S and 5.8S rRNA genes ITS6 5′-GAAGGTGAAGTCGTAACAAGG-3′ and ITS7 5′- AGCGTTCTTCATCGATGTGC-3′ (here called oomycetes ITS1) (Cooke et al., 2000). For the prokaryotic root-associated samples, peptide nucleic acid blockers (PNA BIO, Newbury Park, CA, United States) against plant mitochondria and chloroplasts were added as described previously (Lundberg et al., 2013). Each PCR sample was uniquely barcoded using a double-barcode system (Fadrosh et al., 2014). Phusion high-fidelity DNA polymerase (Thermo Fisher, Waltham Scientific, MA, United States) was used according to manufacturer’s specifications in a final volume of 20 μl. A standardized amount of DNA (5 ng) was added to each PCR mixture. Cycling conditions for prokaryotic and eukaryotic primers were: 98°C for 1 min; 25 cycles of 98°C for 15 s, 65°C for 15 s, and 72°C for 30 s, with a final extension at 72°C for 7 min. For fungal and oomycete primers the following conditions were used: 98°C for 1 min; 35 cycles of 98°C for 30 s, 65°C for 30 s, and 72°C for 30 s, with a final extension at 72°C for 7 min. Ten biological replicates for PCR from rhizosphere and root samples were used. Next Generation Sequencing (NGS) with Illumina MiSeq v3 chemistry 300 base paired-end sequencing (Illumina, San Diego, CA, United States) was performed at the Wellcome Trust Centre for Human Genetics, Oxford, United Kingdom.

Quantitative PCR was performed using iQ SYBR Green Supermix (BioRad, Hercules, CA, United States) using the same DNA samples and primers as above. Reactions were incubated in a CFX96 thermocycler (BioRad) as follows; 95°C for 5 min followed by 40 cycles of 94°C for 15 s, 57°C (16S rRNA) or 64°C (18S rRNA) for 30 s and 72°C for 30 s. NGS data was used to exclude host plant DNA contribution in the soil 18S rRNA (ranging from 14 to 75% of the eukaryotic community obtained using 18S rRNA primers). Hence, if 50% of the 18S rRNA amplicons were identified as originating from the host plant, then the 18S qPCR results were halved. As it is not possible to obtain a titration curve for the environmental DNA and establish an absolute copy number of the gene of interest in a DNA sample, we only compare the relative amplification detection threshold of 16S and 18S rRNA genes against each other. For each DNA sample (5 ng), Ct (cycle threshold) was recorded using 16S and 18S rRNA-specific qPCR, transformed using 2^(-Ct) equation to produce 16S “value” and 18S “value.” These “values” were added together (Supplementary Table S1). Contribution of 16S and 18S rRNA genes was calculated based on the 16S and 18S “values” contribution to the “value” sum. This method can’t be used to measure the total microbial load in a sample and likely suffers from various biases due to a different number of 16S and 18S rRNA operons per genome, DNA isolation efficiency and qPCR amplification specificity. However, these biases will occur in all measured samples and hence sample-to-sample comparison of 16S to 18S rRNA content should be valid.

### Data Analysis

Metagenomic data were analyzed using the Usearch pipeline described previously (Tkacz et al., 2018). MiSeq paired-end reads were aligned using FLASh and quality filtered with fastq_quality_filter producing in a total of 20 million high-quality sequences (Magoc and Salzberg, 2011). Reads matching plant DNA (eukaryotic primers) and its organelles (prokaryotic primers) were removed prior to statistical analysis using a custom-made Linux script (Supplementary Table S1). For each set of samples (prokaryotes, eukaryotes, fungi, and oomycetes) an upper threshold value was set to standardize number of sequences per sample: 10,000 sequences per sample for bacteria, 5,000 for eukaryotes, 3,000 for fungi, and 2,000 for oomycetes library. Reads were processed using custom-made and FASTX-Toolkit Bash and Python scripts in Unix^1^ and clustered into Operational taxonomic units (OTUs) at 97% similarity threshold according to the Usearch pipeline (Edgar, 2010). Prokaryotic and eukaryotic origin OTUs were annotated using 16S/18S rRNA gene database SILVA 123.1 (Quast et al., 2013) and fungal and oomycetes ITS1 OTUs using the GenBank database. The removal of all DNA reads of plant, mitochondrial and chloroplast origin was made by binning all reads (before standardization) into OTUs of 100% similarity annotated against the same SILVA database. All OTUs, for which the best match was a plant entry were scored and their reads removed from the samples. The OTU table (number of reads belonging to each OTU per sample) was uploaded into PRIMER 6.0 software. Data was standardized (a total contribution of all OTUs per sample to be 100%) and square root normalized. Based on this data Principal Coordinates Analysis (PCoA), Principal Component Analysis (PCA) and Canonical analysis of principal coordinates (CAP) plots were constructed and PERMANOVA calculated. Factors shaping the microbial community were statistically assessed using permutation of residuals under a reduced model, sum of squares type I (sequential) (to allow for low number of degree of freedom data to be tested, i.e., fraction has two conditions – rhizosphere and root, ancestry has three conditions) with 9999 permutations using fixed multifactorial design. This PERMANOVA type allows for assessing influence of different factors on the community with the assumption that all these factors are unordered, and samples belonging to different factorial groups are compared against each other (for example, all the plant genotypes are compared irrespectively of their plant species, ancestry class or ploidy). Additionally, a multifactorial, nested design PERMANOVA (type I and type III) was run in order to test for factorial significance using plant genotypes nested in plant species, which in turn were nested in ancestry class. Such an approach is more restrictive than not-nested approach as genotypes are compared only against each other among the same plant species and plant species are compared only among the same ancestry class. For a confirmation of the findings, a high-rank nested design was also tested for prokaryotic community, where all the factors are nested inside fractions factor (Anderson et al., 2008). The raw PERMANOVA output can be found in Supplementary Table S1. The same data and factors were used for CAP plotting. CAP in comparison to PCoA plots enhances the sample separation based on selected factors. Pearson’s correlation was calculated in Excel using CORREL function.

For Venn diagram construction, only OTUs present in all lines (two lines for prokaryotes, four lines for eukaryotes, fungi, and Oomycetes, and present in at least 25% of the biological replicates for each of these lines) of the same species were considered as stably associated with a given wheat species. Bar plot figures were created in PRISM 8. This software was also used to calculate ANOVA and pair-wise *t*-tests with Bonferroni correction for the qPCR results and to test significance of phylum level (prokaryotes and eukaryotes) and genera level (fungi and Oomycetes) changes between different fractions. We consider OTUs matching to orders of Glomerales, Diversisporales, Archaeosporales, and Paraglomorerales as members of Glomeromycetes class (Redecker et al., 2013).

## Results

### Control Over Rhizosphere Eukaryote/Prokaryote Abundance Ratio Is Conserved Across Wheat Species and Their Progenitors

We observed from qPCR targeting eukaryotic 18S and prokaryotic 16S rRNA that none of the rhizospheres of the wheat species examined in this study (Table 1) differs from each other in proportion of eukaryotes to prokaryotes (Supplementary Figure S1). In each case, the ratio is similar to that of the original soil or bulk soil control (Supplementary Figure S1) (based on pair-wise *t*-tests with Bonferroni correction). Values obtained in this work using 16S and 18S rRNA-specific qPCR (corrected for the host plant DNA abundance) are similar to those we previously reported from RNA-Seq-based data (Turner et al., 2013). RNA-Seq data gave the proportion of eukaryotes to prokaryotes in the bulk soil as 2.8% and was similar to that from the rhizosphere of *T. aestivum* cv. Paragon (3.3%). In this work, using qPCR we report similar values; 5.4% eukaryotes in bulk soil with 6.8% in the rhizosphere of *T. aestivum* cv. Paragon. Other wheat species have similar ratio values to that of cultivar Paragon (approx. 6.6% averaged across all wheat lines) (Supplementary Figure S1) with none reaching the higher levels of approx. 21% observed in pea and oat (Turner et al., 2013). These results indicate that maintenance of a low ratio of eukaryotes to prokaryotes in the soil surrounding their roots is conserved across modern wheat, its progenitors and crosses, and is independent of ploidy and any given history of artificial selection.

### Prokaryotic, Eukaryotic, Fungal, and Oomycete Community Members Are Shared Across Wheat Species

Since the rhizospheres of the wheat species analyzed show no overall change in their eukaryotic/prokaryotic ratio, we, therefore, investigated differences in the community structure of these two life domains. For consistency, we initially focused on the rhizosphere prokaryotic and eukaryotic communities, later expanding our study into fungal and oomycetes communities. Based on other studies (Bulgarelli et al., 2012) we suspected that the largest shifts in community structure may occur within the microbiota of the root fraction (both on the surface and inside roots), rather than in the rhizosphere. For this reason, we examined the prokaryotic community in both niches for two lines of each of five different wheats (Table 1). We did not attempt to examine more closely the root microbiota for other microbial life domains as it is extremely difficult to remove the host plant DNA sufficiently to run effective PCR-amplification of eukaryotic 18S rRNA.

**FIGURE 2:**
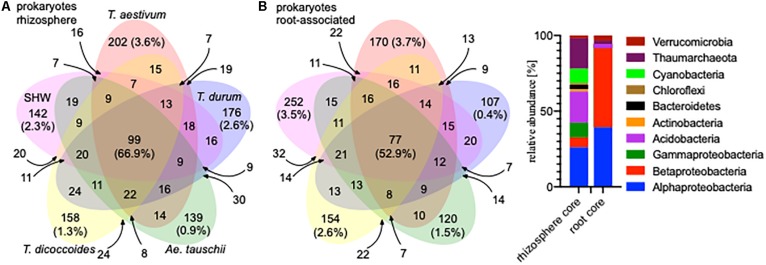
Venn diagrams of the prokaryotic microbiota from different wheat species in **(A)** rhizosphere and **(B)** root-associated niches showing the number and relative abundance of prokaryotic OTUs. Only OTUs that were found in both lines representing a species were analyzed. For the small overlapping regions, the OTU numbers are indicated with arrows. Core microbiota profile of the rhizosphere and root-associated community is presented.

A large proportion of the microbiota is shared between all analyzed wheat species; 99 OTUs (66.9% of the total community abundance) in the rhizosphere (belonging to Acidobacteria, Thaumarchaeota and to Proteobacteria) and 77 OTUs (equating to 52.9%) in the root-associated fraction (belonging predominantly to Alpha- and Betaproteobacteria) (Figure 2). Indeed, individual wheat species show a relatively weak selection pressure on the prokaryotic microbiota, as only approx. 2% of the total community (0.9–3.6% for the rhizosphere microbiota and 0.4–3.7% for the root microbiota abundance) was classified as being specific (found in a core set of a given wheat species microbiota – i.e., present in all lines, but not found in a core of any other wheat species) for a single wheat species, albeit these small microbiotas consist of many different OTUs. In many cases (apart from *T. durum*) there is stronger selection by the roots compared to the rhizosphere (Figure 2) and the root-associated samples from SHW and *T. aestivum* plants especially, are colonized by unique microbiota, both in terms of OTUs number and their relative abundance (252 OTUs making up 3.5% abundance and 170 OTUs totaling 3.7% abundance for SHW and *T. aestivum*, respectively, Figure 2).

General eukaryotic, as well as more specific fungal and oomycete groups, were also analyzed in the same way, however, due to problems from unwanted amplification of plant material, only the rhizosphere fraction was characterized (Supplementary Figure S2). The eukaryotic core (shared by all) consists of 242 OTUs, which make up 94.8% of the community abundance (belonging predominantly to Ascomycota). The fungal core consists of 185 OTUs making up 96.5% of the community (many minor genera) and the oomycetes core consists of only 34 OTUs making up 98.4% of the community (almost only *Pythium* spp.) (Supplementary Figure S2).

### Wheat Selects Three Distinct Prokaryotic and Eukaryotic Community Assemblies

**FIGURE 3:**
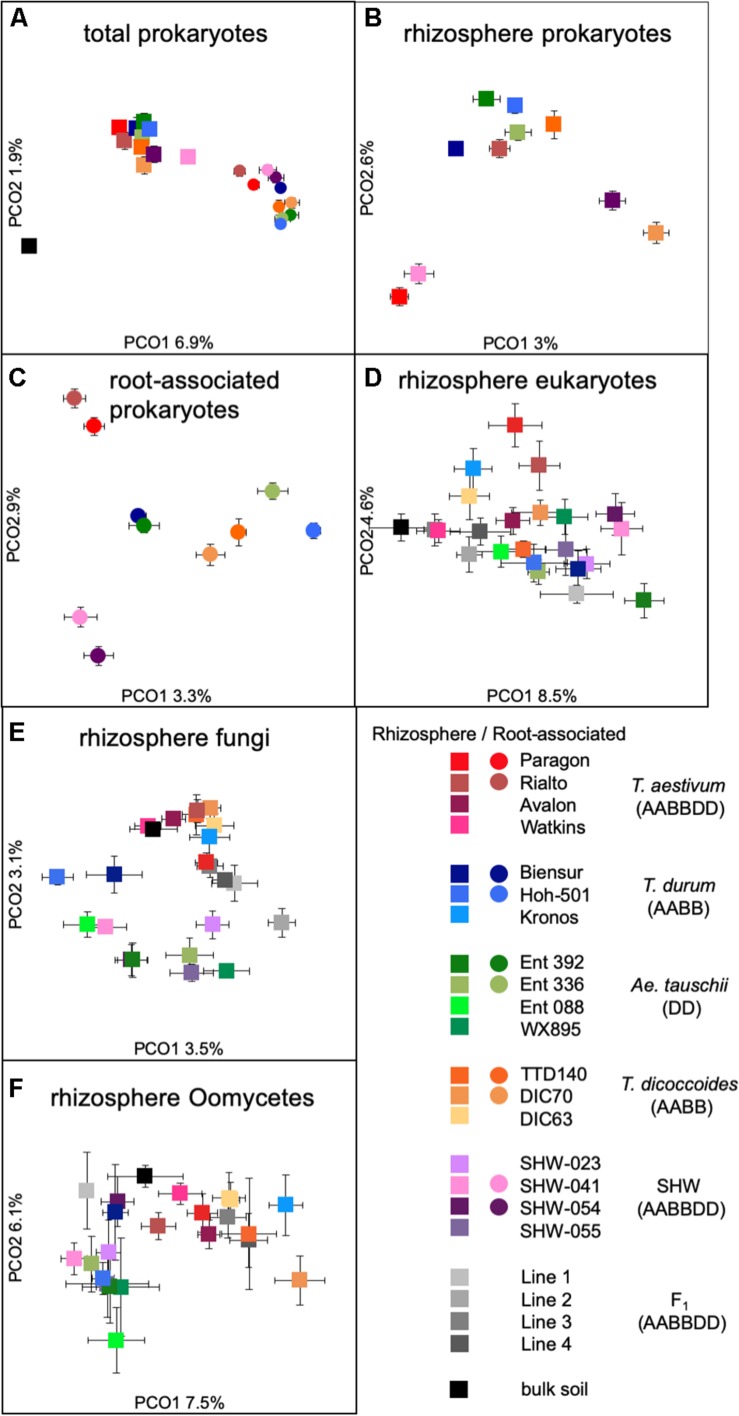
PCoA plots of **(A)** total prokaryotic community, **(B)** prokaryotic rhizosphere community, **(C)** root-associated community, **(D)** eukaryotic rhizosphere community, **(E)** fungal rhizosphere community, and **(F)** Oomycetes rhizosphere community. Data points represent averaged (mean) location of all individual samples belonging to a given group, while error bars represent standard error.

While there is a large common core microbiota, we wished to investigate whether plants influence prokaryotic and eukaryotic organisms that are less abundant. Data binned into OTUs was square-rooted to suppress the importance of dominant species and focus on the less common microbiota. Transformed OTU-based data was visualized using PCoA plots. Samples representing the structure of prokaryotic communities from bulk soil, rhizosphere and root (i.e., niche) align along the first PCoA axis (Figure 3A) indicating that it is the niche, rather than plant species, which is the major factor shaping these communities. In order to statistically test our observations, we have run multifactorial PERMANOVA (Supplementary Figure S3 and Supplementary Table S1). Fractions (niche) is the strongest factor (pseudo-F = 10.5, *P* < 0.0001), separating the prokaryotic community, ploidy, ancestry class (modern wheat, wild wheat, and synthetic hybrids) and plant genotype factors have a weaker, yet significant influence (pseudo-F of 1.6, 2.1, and 1.2, respectively, *P* < 0.0001). From all the factors tested, only plant species failed to reach significance with *P* = 0.06.

We have also run nested multifactorial PERMANOVA of type I and type III as well as high-rank nested design where all factors are nested inside fractions. Plant genotypes factor consistently show a significant influence on the total community or separately rhizosphere or root community, even if normally other factors failed to reach significance in these more restrictive statistical tests (Supplementary Table S1).

When analyzing the microbiota separately for each fraction, we observe that all these factors have a similar weak, yet significant influence and only plant species factor seems not to be important for the root samples.

There is a strong general plant effect (rhizosphere vs. root) on the prokaryotic community, as the root microbiota is clearly different from that of the rhizosphere and bulk soil (Figure 3A). The overall plant species influence is similar in the rhizosphere and root (pseudo-F of 1.45 and 1.40 for rhizosphere and root, respectively, *P* < 0.0001). PCoA plot of the rhizosphere community separates Paragon and one of the SHW lines from the rest of the plant microbiota (Figure 3B). Despite, the fact that PERMANOVA is able to separate the community based on the plant ploidy, ancestry class, plant species and genotype (Supplementary Figure S3) it is much easier to see these relations in CAP analysis (Supplementary Figure S4) than from the PCoA. We decided to focus on the root microbiota, which, analysis shows, can be split into three groups (Figure 3C): Group 1, influenced by *T. aestivum* cv. Paragon and Rialto lines, Group 2, influenced by SHW (SHW-041 and SHW-054), with a looser Group 3, which clusters away from the other two, influenced by wild grasses (*T. dicoccoides*, *A. tauschii*) and *T. durum* (PERMANOVA for prokaryotes with three clusters of plant genotypes; pseudo-F 1.9, *P* < 0.0001, and high-rank PERMANOVA pseudo-F 1.56, *P* < 0.0001). The influence of factors used in PERMANOVA was visualized with CAP plots. CAP plots clearly separate both rhizosphere and root samples based on the selected factors (Supplementary Figure S4).

A similar plant genomic effect is also observed for the rhizosphere eukaryotic community (PERMANOVA for eukaryotes with three clusters of plant genotypes; pseudo-F 3.1, *P* < 0.0001, for all plant genotypes; pseudo-F 1.4, *P* < 0.0001) (Figure 3D, Supplementary Figure S3 and Supplementary Table S1). Moreover, *A. tauschii* and its SHW lines, have an effect on the fungal (Figure 3E) and oomycete plants (PERMANOVA for oomycetes with three clusters – SHW, *A. tauschii* and other species; pseudo-F 2.5, *P* < 0.0001) (Figure 3F) community where they form a separate cluster on PCoA plots, away from other samples. For fungi, the F_1_ plants (cross between SHW and *T. aestivum*) also form a cluster, close to that of *A. tauschii*-SHW suggesting a similar fungal community shared between these plants (PERMANOVA for fungi with four clusters – SHW, *A. tauschii*, F_1_ and other species; pseudo-F 2.44, *P* < 0.0001) (Figure 3D). Observations from PCoA visualization and PERMANOVA statistics correspond with the Venn-based analysis, which shows *T. aestivum* and SHW lines were colonized by specific prokaryotic OTUs, while individual plant species exerted a weaker (yet, significant according to PERMANOVA) effect on eukaryotic, fungal and oomycetes communities (Figure 2 and Supplementary Figure S2). Nested design PERMANOVA revealed that only plant genotypes factor is significant in separating rhizosphere eukaryotic, fungal and Oomycetes community. Influence of factors separating the eukaryotic, fungal, and oomycetes community were further validated using CAP analysis. CAP plots clearly separate communities based on the host plant ploidy, ancestral class, plant species, and genotype (Supplementary Figure S5).

### Wheat Plants Are Colonized by Proteobacteria, and, in Addition, *T. aestivum* Is Specifically Colonized by Cyanobacteria

**FIGURE 4:**
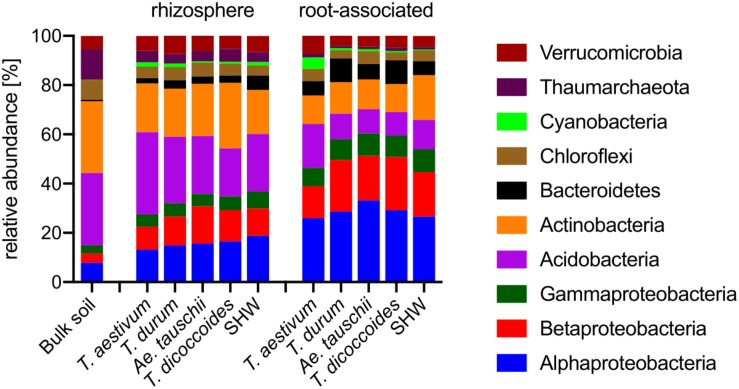
Phylogenetic annotation of rhizosphere and root-associated OTUs found for each wheat species. Bars represent averaged relative abundance of each phyla for each wheat species. bulk soil *n* = 8, rhizosphere *n* = 24, 23, 18, 20, 24, root *n* = 23, 16, 15, 18, 23 biological replicates for each condition.

Roots of wheat plants and their rhizospheres are colonized predominantly by Proteobacteria (Figure 4). While this phylum (Alpha-, Beta-, and Gammaproteobacteria) contributes only 14.9% in bulk soil, it rises to approx. 33.6% in the rhizospheres and reaches approx. 71.1% in the root microbiome (*t*-test results: bulk soil vs. rhizosphere *P* < 0.0001, and rhizosphere vs. root *P* < 0.0001). In contrast, the abundance of phyla common in soil, such as Actinobacteria and Acidobacteria are largely reduced, especially in the root-associated microbiota (*t*-test results for Actinobacteria bulk soil vs. rhizosphere *P* < 0.0001 and *P* = 0.29, and rhizosphere vs. root *P* < 0.0001 and *P* < 0.0001, for Actinobacteria and Acidobacteria, respectively) (Figure 4).

*Triticum aestivum* cv. Paragon and Rialto have a noticeably different abundance of prokaryotic phyla compared to other wheat species, with an increase in Cyanobacteria belonging to two genera; *Nostoc* and *Cylindrospermum* in association with roots up to 3.9 and 5.4%, respectively (*t*-test for root abundance of Cyanobacteria of *T. aestivum* vs. all other species *P* < 0.0001) (Figure 4 and Supplementary Figure S6). Cyanobacteria abundance in *T. aestivum* root is eight times higher than in other wheat species. While statistical tests (Supplementary Table S1) are able to point to many other more subtle phyla abundance shifts in the rhizosphere and roots, no other phyla is even doubled in its abundance for any given species (against average abundance of other species).

**FIGURE 5:**
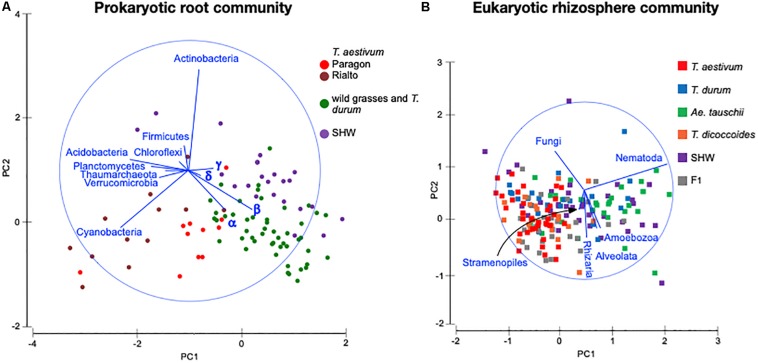
PCA plots of **(A)** prokaryotic root community and **(B)** eukaryotic rhizosphere community at the phylum level. Microbiota taxa are used as factors separating plants microbiota structure. The longer the line representing microbial taxa is, the strong effect it has for the sample differentiation. Samples located according to the lines’ directions are influenced by a particular taxon. α – alpha-, β – beta-, γ – gamma-, δ – Deltaproteobacteria.

Identification of the relative influence of individual factors, such as phyla abundance, on predefined groups of samples, e.g., the three wheat groups initially indicated by PCoA plot (Figure 3C), can be further investigated by PCA analysis. The PCA plot (Figure 5A) shows clearly the importance of Cyanobacteria in the *T. aestivum* community, especially for cultivar Rialto. Proteobacteria, especially sub-phyla Alpha- and Beta-, were important for other wheat lines (Figure 5A).

### *A. tauschii*-Derived D Genome Increases the Abundance of Nematoda and Glomeromycetes

**FIGURE 6:**
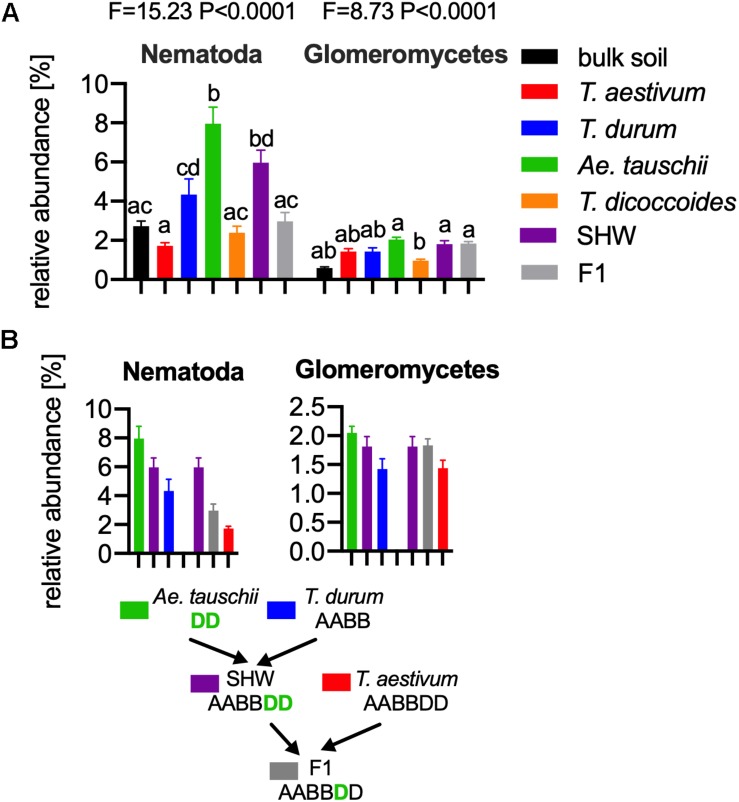
**(A)** The relative abundance of Nematoda (left) and Glomeromycetes (right) in the microbial eukaryotic community. ANOVA F and *P* values are provided, and the letters present the result of pair-wise *t*-test with Bonferroni correction. **(B)** *A. tauschii* derived D genome increases the influence of these taxa, while the artificially selected D does not have such an effect, for panels **(A,B)** error bars represent standard error. Arrows indicate the hybridization and crosses conducted in this study. Different letters reflect significant difference between datasets in pair-wise *t*-test.

As the general eukaryotic phylogenetic profile is conserved between all wheat species examined and dominated by fungi (Supplementary Figure S7A), we decided to use PCA analysis to visualize the more subtle differences between the wheat species (Figure 5B). The striking observation is the strong selection for Nematoda in the rhizosphere of *A. tauschii*, SHW and some F_1_ hybrids (Figure 5B). We plotted the relative abundance of Nematoda for each wheat species and observe a clear relationship between the presence of the wild D-genome of *A. tauschii* (wild D) and the abundance of Nematoda in the rhizosphere. Nematoda reads contribute to up approx. 8% of the *A. tauschii* rhizosphere sequencing output compared to approx. 4% in *T. durum* and only 2% in *T. aestivum*, *T. dicoccoides* and bulk soil (Figure 6A). Crucially, the high abundance of Nematoda is observed in wheat lines crossed with *A. tauschii* i.e., those with wild D present (Table 1); SHW lines have an average abundance of Nematoda (approx. 6%), between that of *A. tauschii* (approx. 8%) (but not statistically significant difference) and *T. durum* (approx. 4%) (significantly different). F_1_ crosses between SHW and *T. aestivum* show abundance of approx. 4% (Figure 6B). The modern bread wheat, *T. aestivum*, strongly suppresses the presence of Nematoda, as its abundance in the rhizosphere of these plants is as low as in bulk soil and wild plants without D genome (*T. dicoccoides*) or its crosses (F_1_ lines) (Figure 6A).

In order to find if there are other eukaryotic taxa whose abundance in the rhizosphere is also influenced by the wild D genome, we ran bivariate correlation (Pearson’s *r*) of Nematoda abundance against other eukaryotic taxonomic groups. Glomeromycetes, (a group of fungi able to provide plants with a means of uptake of inorganic P) relatively abundant taxa showed a strong correlation (*r* = 0.73) to Nematoda presence. It has previously been shown experimentally that *Aegilops* are highly colonized by these fungi compared with *Triticum* species (Kapulnik and Kushnir, 1991). In our experiments, the maximum relative abundance of Glomeromycetes in the rhizosphere was approx. 2%, while in bulk soil it was approx. 0.5% (Figure 6A and Supplementary Figure S7A). We observed a clear influence of the wild D genome in supporting Glomeromycetes abundance in the rhizosphere as both SHW and F_1_ hybrids containing *A. tauschii*-derived D genome shows a statistically significant higher abundance of these fungi than species completely lacking the D genome (*T. dicoccoides*) (Figure 6B). A similar observation can be done using fungal ITS1 analysis, where an elevated abundance of *Glomus* genus, the most abundant Glomeromycetes genus can be found in plants with wild D genome (*A. tauschii* with abundance of 1.4% of the relative fungal community). The lowest *Glomus* relative abundance was found in *T. dicoccoides* (0.6%), *T. durum* (0.8%) both lacking the D genome, *T. aestivum* (0.9%) containing domesticated D genome and the bulk soil control (0.2%) (Supplementary Figures S7A,B for comparison).

We have further analyzed the Glomeromycetes and Nematoda communities in order to establish either plant genotypes influence not only their rhizosphere abundance but also their structure. We have constructed PCoA plots, using only OTUs that were annotated to the selected group (Glomeromycetes and Nematoda) (Supplementary Figure S8). Glomeromycetes community structure may be weakly shifted in *A. tauschii* and SHW rhizosphere, especially compared to the bulk soil (Supplementary Figures S8A,B), while the Nematoda community structure is shared across all the rhizospheres and is similar to the bulk soil (Supplementary Figures S8C,D).

It is important to note that we were probably unable to sample all of the Nematoda community as some of the species are just too large to be captured in the rhizosphere samples. However, many Nematoda species of 1 mm length or less could adhere to soil or roots and be collected through shaking and vortexing roots.

### Wheat Species Do Not Strongly Select for Other Major Fungal and Oomycetes Groups in the Rhizosphere

The fungal-specific community profile based on sequencing of ITS1 genomic region indicates that *Mortierella*, *Verticillium*, *Fusarium*, *Cryptococcus*, and *Clavaria* are dominant soil and rhizosphere genera. Apart from *Glomus*, which rhizosphere relative abundance can be correlated with host wheat wild D genome presence, there are no other taxonomical major changes between bulk soil and rhizospheres (however, *A. tauschii* rhizosphere statistically differentiate abundance of a few fungal taxa). The soil and rhizosphere oomycetes specific profiles are dominated by *Pythium* with a slight rhizosphere selection for *Phytophthora* genus comparing to the bulk soil (Supplementary Figures S7B,C).

## Discussion

We have characterized the influence of modern bread and pasta wheat lines, their wild ancestors and synthetic hybrid crosses on the structure of the soil microbiota that they support (Table 1 and Figure 1). We have demonstrated that none of these plants changes the ratio of microbial eukaryotes to prokaryotes isolated from their rhizospheres (Supplementary Figure S1), unlike in soil-grown pea and oat (Turner et al., 2013). One possible explanation is that wheat does not allow fungi to dominate the rhizosphere and colonize their roots, which may be a protective mechanism against soil-borne fungal disease. Crucially, this ability is deeply rooted in the wheat genome rather than being an unforeseen consequence of plant domestication and breeding.

We have observed three distinct groups of prokaryotic and eukaryotic microbiota from examining wheat; one for bread wheat, a second for SHW and the third for the wild grasses and pasta wheat (Figure 3). As bread wheat (AABBDD) and SHW (AABBDD) form separate interactions from each other and from all other wheat plants examined, we speculate that one of the factors controlling the microbiota structure is plant domestication and genomic content, rather than ploidy level, *per se*. Our findings using wheat wild progenitors, modern cultivars and their hybrids mostly agree with results of maize rhizosphere study, where teosinte, inbred and modern hybrids select for three distinct prokaryotic and fungal communities (Brisson et al., 2019).

Wheat species, despite possessing a substantial core microbiota shared between all the examined lines (Figure 2), are colonized by different bacterial phyla. Bread wheat supports a relatively high abundance of Cyanobacteria. Cyanobacteria are able to establish symbioses with a range of plant species (Bergman et al., 1996; Adams and Duggan, 2008). *T. aestivum* cultivars Rialto and Paragon were predominantly colonized by *Nostoc* spp. and *Cylindrospermum* spp. (Figures 4, 5 and Supplementary Figure S6) previously shown to fix atmospheric N in rice roots (Nilsson et al., 2002). We did not establish if our plants interacted with Cyanobacteria in order to obtain additional N, however, we do show that modern bread wheat is more capable of such interactions. We speculate that the presence of Cyanobacteria in roots may have been overlooked as it is common to use chloroplast and Cyanobacteria-selective primers for PCR-based assays, or bioinformatically remove Cyanobacteria reads from the metagenomic output (as a potential plant organelle contamination) or focus solely on plant-rhizosphere interactions (Kawasaki et al., 2016; Gdanetz and Trail, 2017; Liu et al., 2017; Kavamura et al., 2018). Following observations made on barley, lettuce, bean and *Arabidopsis* examining modern and wild relatives (Perez-Jaramillo et al., 2015) we do observe an elevated abundance of Bacteroidetes in the roots of wild *T. dicoccoides* but also its domesticated species *T. durum* (Figure 4, Supplementary Figure S6 and Supplementary Table S1).

Plant genomic content plays a major role in the interactions with soil microbial eukaryotes. Genes present on the D genome of *A. tauschii*, a wild goat grass progenitor of modern bread wheat (Figure 1), strongly select for Glomeromycetes and Nematoda. Crucially, we show that by crossing *A. tauschii* with other species, both the Glomeromycetes and Nematoda presence increases in the rhizosphere of progeny plants (Figure 6). Previous studies showed that wheat containing the *A. tauschii* D genome was better colonized by mycorrhizal fungi compared to those with solely A or B genomes (Kapulnik and Kushnir, 1991). Moreover, the D genome of *A. tauschii* has been identified as a determinant of mycorrhiza colonization of its progeny hexaploid wheat lines (Hetrick et al., 1993). We show that the D genome allows the plants to select some of the eukaryotic groups. While an increased interaction may be positive as in case of the mycorrhizal fungi as Glomeromycetes, it may also be potentially detrimental, as in the case of Nematoda. Nematoda contains many economically important plant pathogens (Jones et al., 2013), however, it is not possible to determine if the Nematoda of this study are truly plants pathogens as a fragment of 18S rRNA gene sequence does not allow for a genus-specific identification. Naturally, many Nematoda are common soil organisms and maybe even increasing plant biomass by P and N nutrients cycling in the soil (Gebremikael et al., 2016). However, we can hypothesize that the ability of the plant to select Nematoda in its rhizosphere may have been one of the unseen factors in plant selection and domestication. The highly selected modern bread wheat *T. aestivum*, which does contain a historically introduced *A. tauschii*-derived D genome, added prior to later extensive domestication, strongly suppresses Nematoda, even compared to its abundance in bulk soil. It is possible that Glomeromycetes and Nematoda interact with each other (Yang et al., 2014), for example, it has been observed that mycorrhiza fungi, both directly and by priming plant defense, is able to reduce Nematoda gall formation (Hao et al., 2012). However, our experimental setup does not allow us to make any conclusions about their interactions above stated that their relative abundance seem to be correlated.

In summary, we suggest that attention should be given to the root and rhizosphere microbiota, an often-overlooked byproduct of the plant breeding process. Through observation of this important biological reservoir during the process of plant breeding, new plant varieties can be obtained with increased interactions with beneficial microbes and the ability to suppress unwanted pathogens.

## Data Availability Statement

The datasets generated for this study can be found in the European Nucleotide Archive (ENA) under the accession numbers PRJEB27145 and PRJEB14314. The list of samples used and the code to analyse the data is provided in Supplementary Table S1. Wheat seeds may be obtained through JIC Germplasm Resource Unit (www.seedstor.ac.uk) or by contact with the corresponding author.

## Author Contributions

PP conceptualized the study. AT and PP worked on the Methodology. AT, FP, TT, and EB carried out the investigation. JS, PH, AG, and CU were responsible for the resources. AT, FP, and TT wrote the original draft of the manuscript. AT, FP, JC, and DE worked on the visualization. AT and PP supervised the study.

## Conflict of Interest

The authors declare that the research was conducted in the absence of any commercial or financial relationships that could be construed as a potential conflict of interest.

## Footnotes

1 http://hannonlab.cshl.edu/fastx_toolkit/
