# Supplementary figures and images for: Agricultural Selection of Wheat Has Been Shaped by Plant-Microbe Interactions
Source: Front Microbiol. 2020 Feb 6;11:132. doi: 10.3389/fmicb.2020.00132 (PMC7015950; doi:10.3389/fmicb.2020.00132)

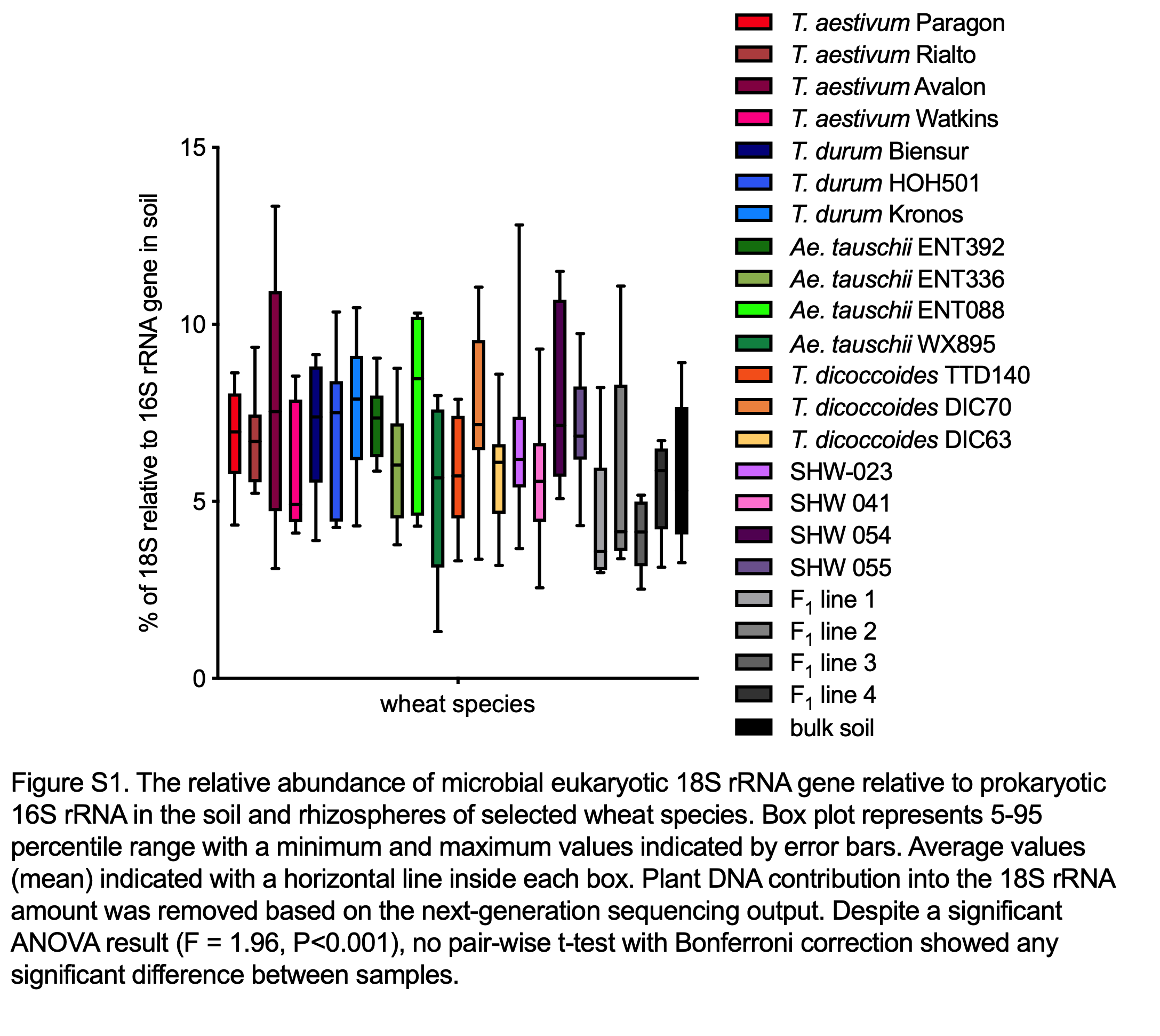

Supplement: Supplementary file 1 [file Image_1.TIFF]

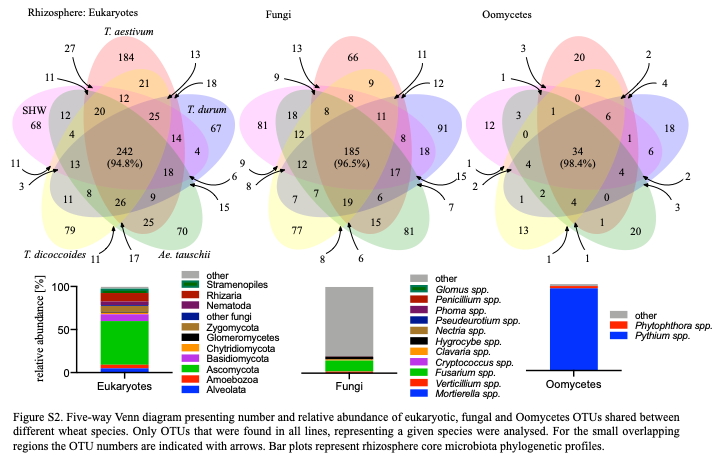

Supplement: Supplementary file 2 [file Image_2.TIFF]

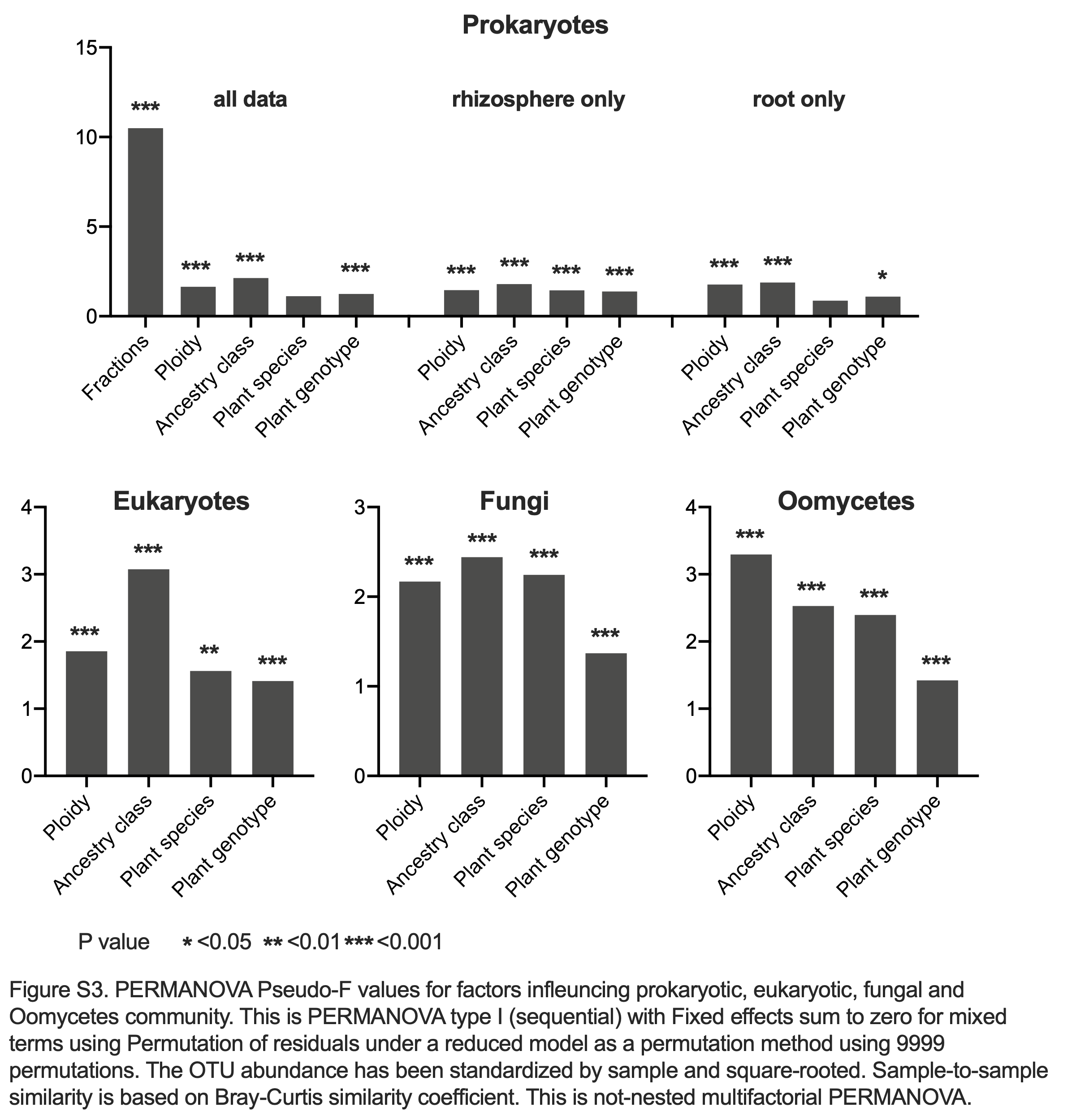

Supplement: Supplementary file 3 [file Image_3.TIFF]

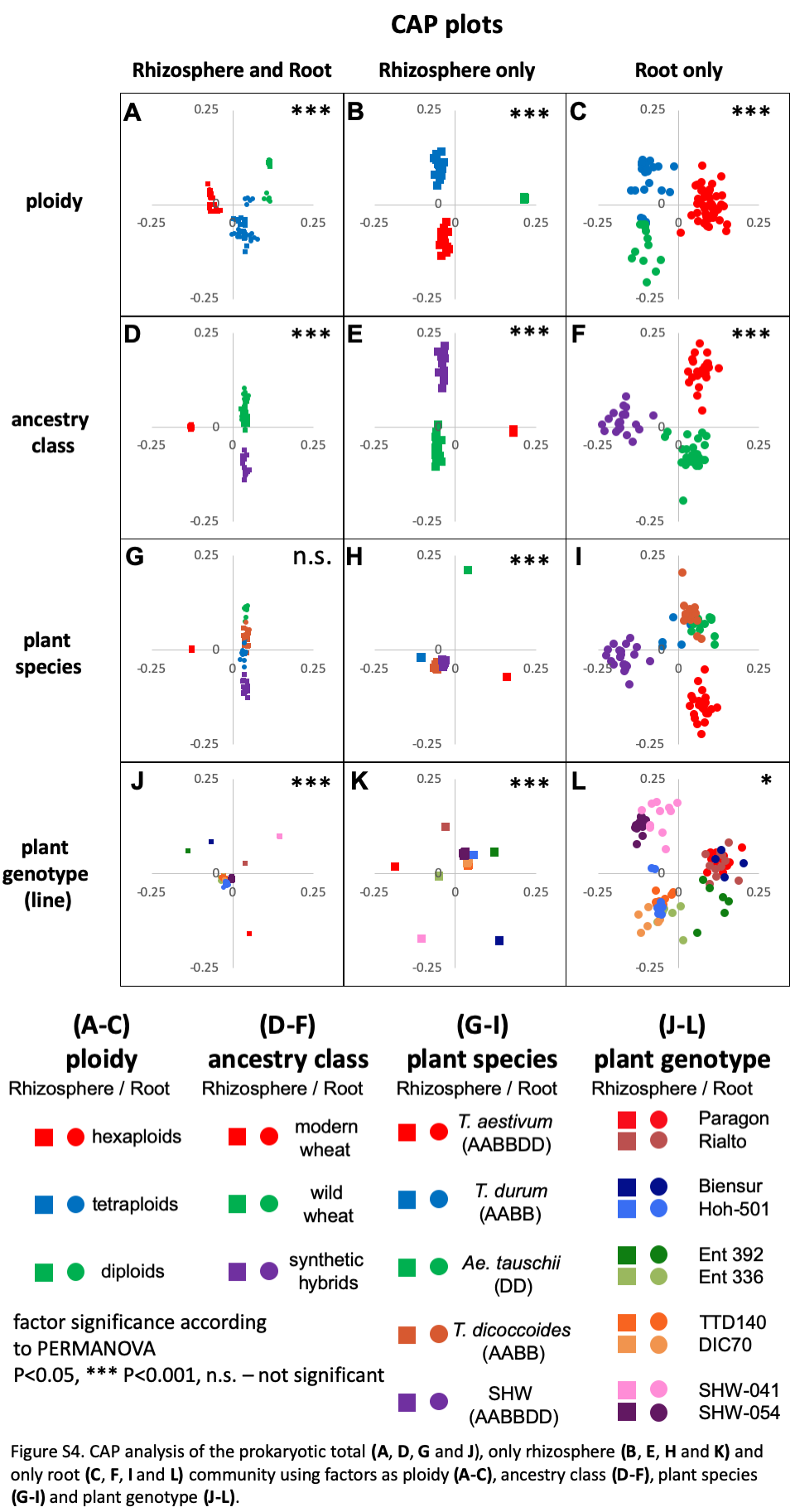

Supplement: Supplementary file 4 [file Image_4.TIFF]

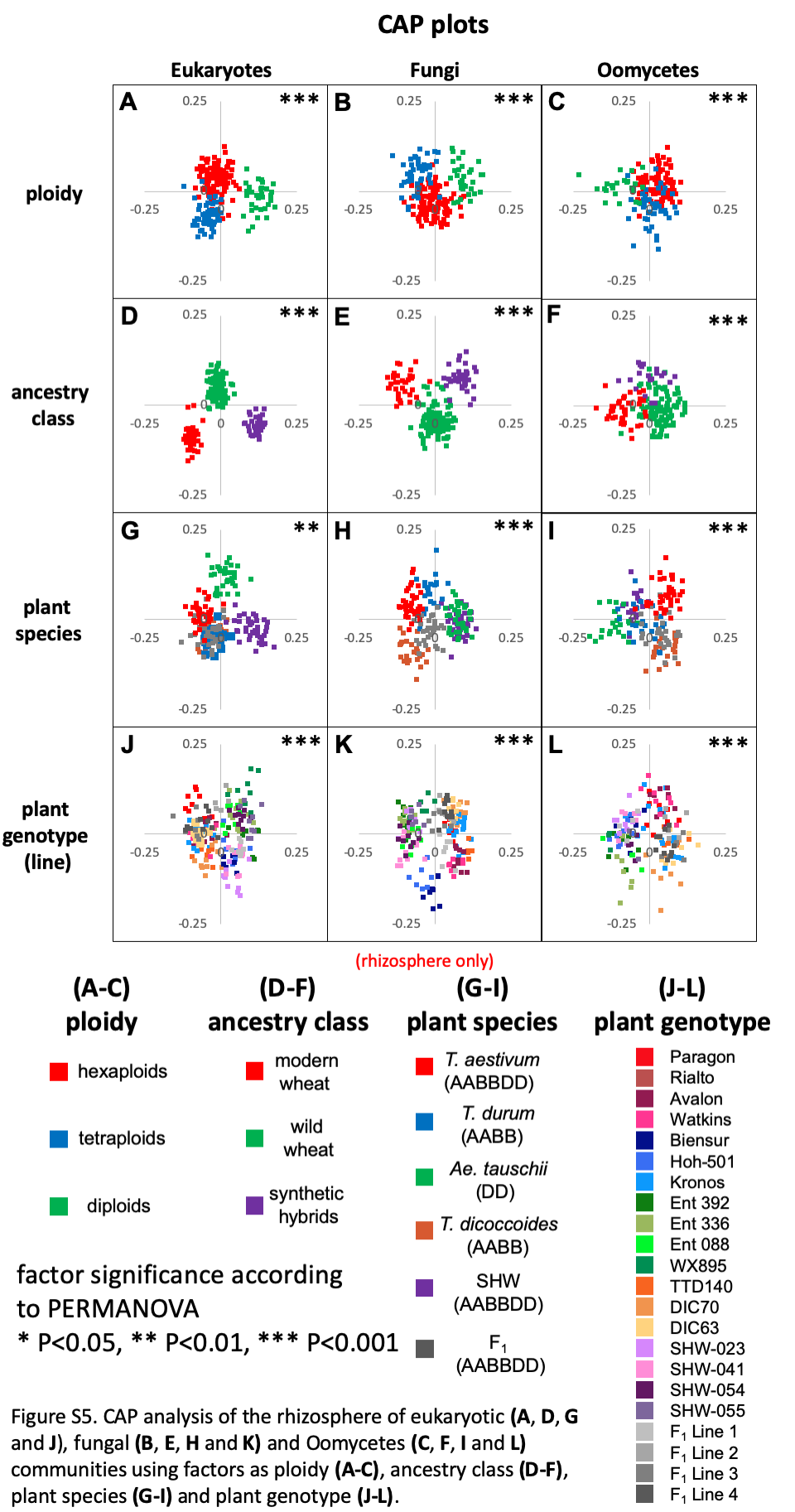

Supplement: Supplementary file 5 [file Image_5.TIFF]

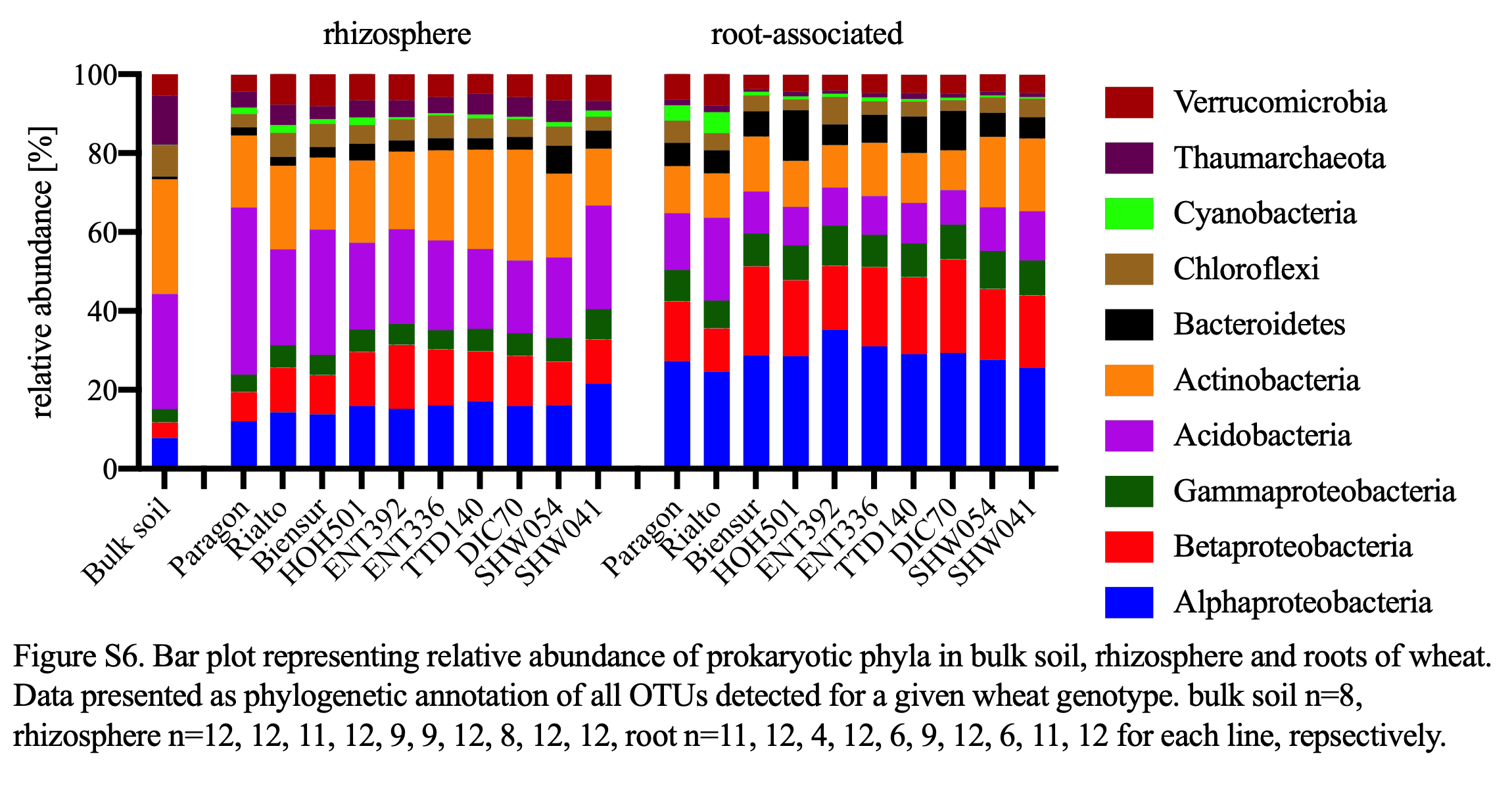

Supplement: Supplementary file 6 [file Image_6.TIFF]

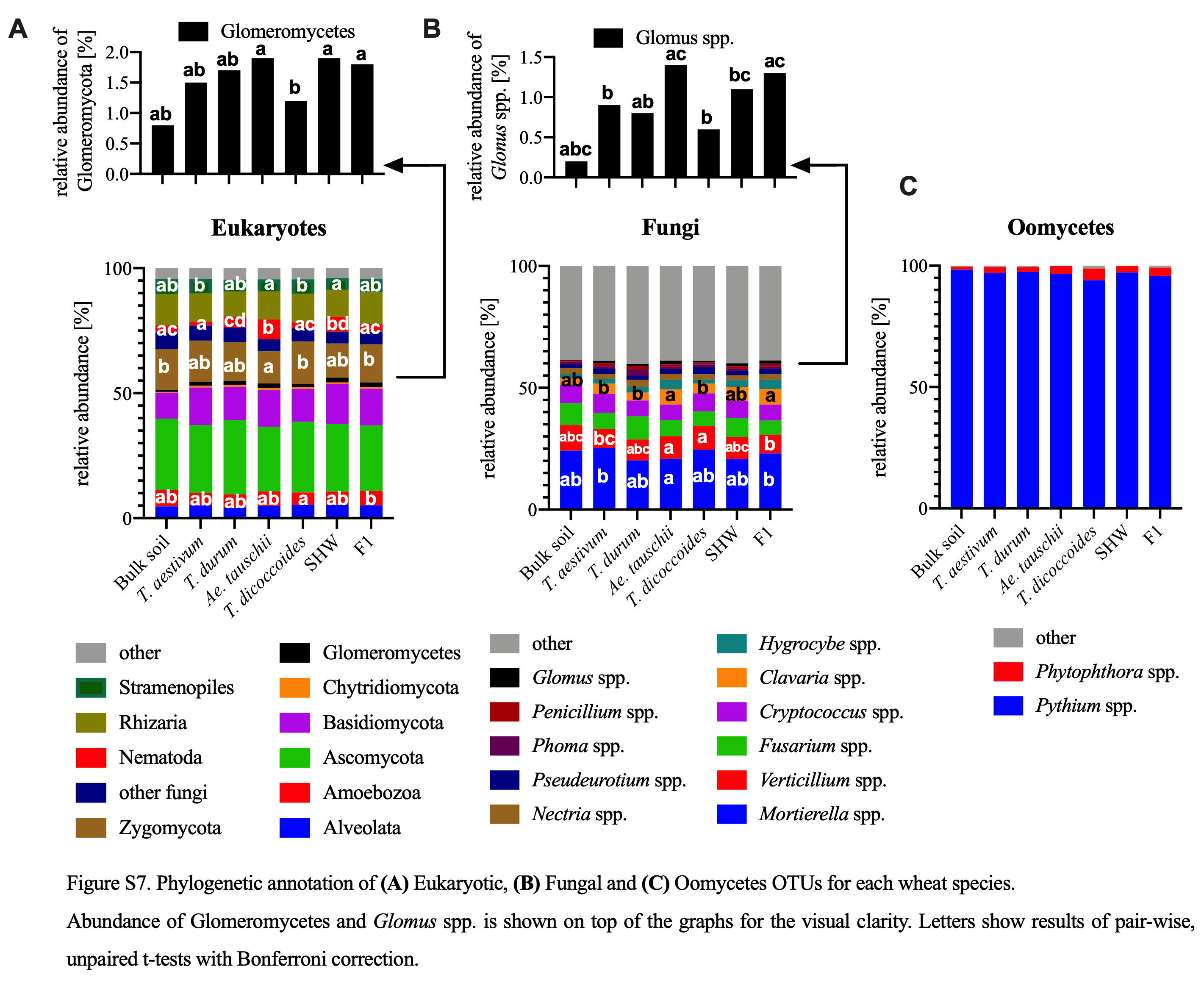

Supplement: Supplementary file 7 [file Image_7.TIFF]

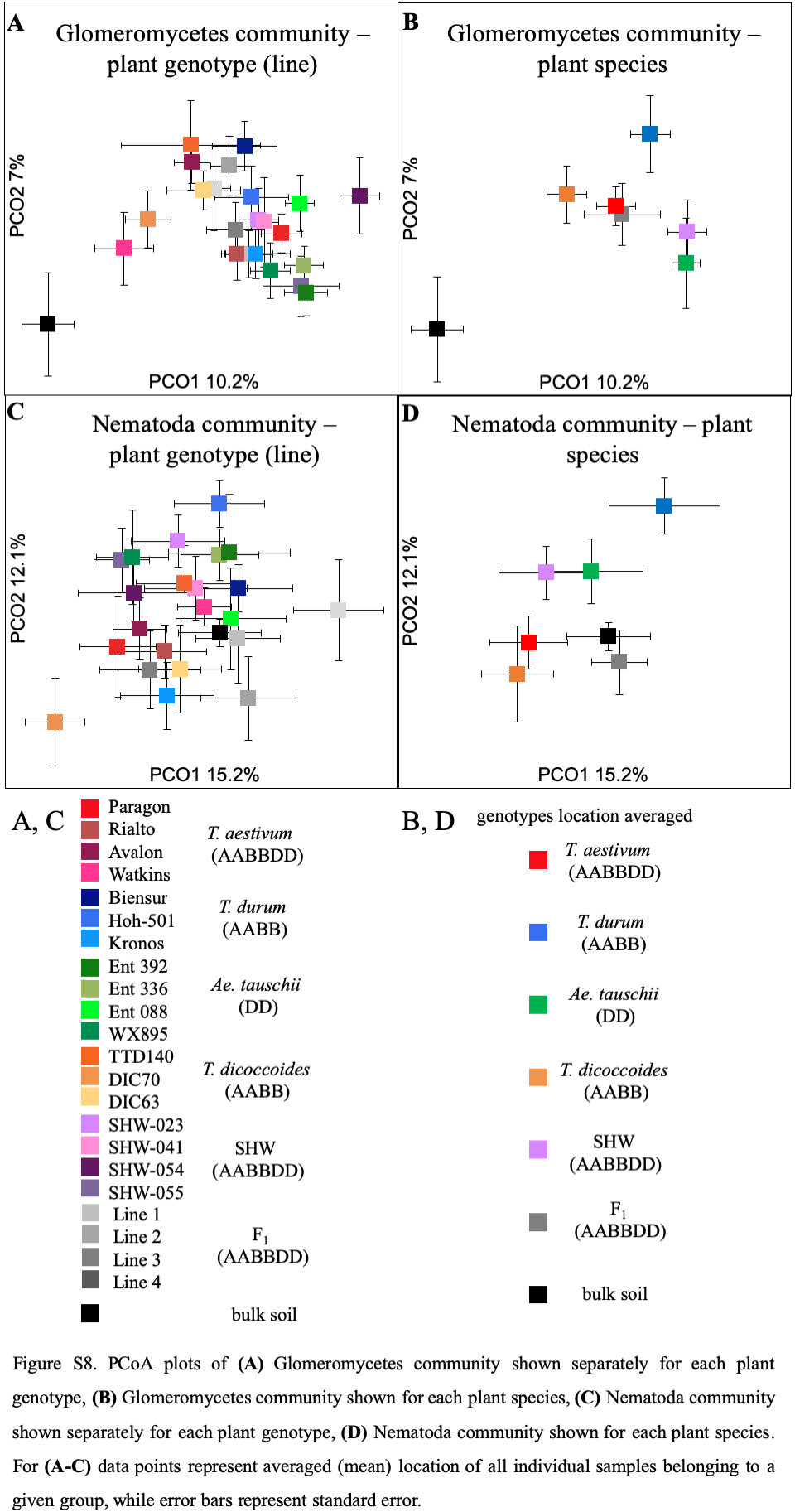

Supplement: Supplementary file 8 [file Image_8.TIFF]
